# TGM2 inhibits the proliferation, migration and tumorigenesis of MDCK cells

**DOI:** 10.1371/journal.pone.0285136

**Published:** 2023-04-28

**Authors:** Zhenyu Qiu, Shouqing Guo, Geng Liu, Mengyuan Pei, Yuejiao Liao, Jiamin Wang, Jiayou Zhang, Di Yang, Zilin Qiao, Zhuo Li, Zhongren Ma, Zhenbin Liu, Xiaoming Yang

**Affiliations:** 1 Northwest Minzu University, Biomedical Research Center, Gansu Tech Innovation Center of Animal Cell, Lanzhou, China; 2 Life Science and Engineering College of Northwest Minzu University, Lanzhou, China; 3 Northwest Minzu University, Biomedical Research Center, Key Laboratory of Biotechnology & Bioengineering of State Ethnic Affairs Commission, Lanzhou, China; 4 National Engineering Technology Research Center of Combined Vaccines, Wuhan, China; 5 China National Biotec Group Company Limited, Beijing, China; Universita degli Studi della Campania Luigi Vanvitelli, ITALY

## Abstract

Madin-Darby canine kidney (MDCK) cells are one of the main cell lines used for influenza vaccine production due to their high virus yield and low mutation resistance. Due to their high tumorigenicity, the safety of vaccines produced from these cells is controversial. TGM2 is a multifunctional protein that plays an important role in the adhesion and migration of cells and is associated with tumor formation. We found that the expression level of TGM2 was significantly up-regulated in low tumorigenic MDCK cells. We first analyzed TGM2-overexpressed and knockout MDCK cells in vitro. Scratch-wound assay and Transwell chamber experiments showed that TGM2 overexpression significantly inhibited the migration and invasion of MDCK cells and significantly reduced their proliferation. TGM2 knockout significantly enhanced cell migration, invasion, and proliferation. The tumorigenesis results in nude mice were consistent with those in vitro. TGM2 knockout significantly enhanced the tumorigenesis rate of MDCK cells in nude mice. We also investigated the effects of TGM2 gene expression on the replication of the H1N1 influenza A virus in MDCK cells. The results showed that TGM2 induced the negative regulation of H1N1 replication. These findings contribute to a comprehensive understanding of the tumor regulation mechanism and biological functions of TGM2.

## 1 Introduction

Influenza is a global disease caused by the influenza virus, resulting in high morbidity and mortality and placing a huge economic burden on society. Vaccination remains by far the best way to prevent an influenza pandemic [1]. The world has the capacity to produce hundreds of millions of doses of vaccine each year, but most (by proportion) influenza vaccines are made from chicken eggs [2]. Compared with the cell culture-based production process, the production of influenza vaccines based on chicken embryos has many limitations, such as long supply time, cumbersome operation process, easy contamination, and potential mutations during the adaptation process for the embryos. The Madin-Darby canine kidney (MDCK) cell line was developed by Madin and Darby in 1958 from kidney tissue obtained from a female cocker spaniel. There are currently two cell-based seasonal influenza vaccines on the market, fluelvax® and SKYCellflu®, both produced from MDCK cells [3]. A key advantage of the MDCK cell line is its broad spectrum, which can be harnessed to efficiently produce influenza strains of multiple subtypes [4]. The use of MDCK cells as viral replication substrates for influenza vaccine production has many advantages, but is still controversial due to the tumorigenicity of the cells [5, 6]. It has been demonstrated that subcutaneous or muscular inoculation of tumor cell lines or their lysates can induce tumors in nude mice. If influenza vaccines are derived from tumorigenic cell lines, oncogenes may be integrated into the human genome after inoculation, posing a potential threat to human health. Effective evaluation of the tumor-forming and tumorigenic properties of MDCK cells to ensure the safety of the cell line for the production of influenza vaccine is the focus of current research.

Transglutaminase (TGM)-2 is a ubiquitous Ca^2+^-dependent protein crosslinking enzyme that plays an important role in wound healing, fiber formation, apoptosis, inflammatory response, cell cycle control, tissue formation, and protection from infection [7, 8]. As a member of the TGM protein family, it has the ability to covalently cross-link substrates [9]. TGM2 can stabilize the extracellular matrix (ECM) through its protein cross-linking activity and also plays an important role in cell survival as a cell adhesion molecule [10]. In addition to participating in the regulation of ECM adhesion, TGM2 is involved in tumor formation. Studies have shown that TGM2 is involved in the occurrence of cancer, and the expression of TGM2 in melanoma, pancreatic cancer, ovarian cancer, and other tumors is higher than that in normal tissues [11–13]. In these cancer cells, up-regulation of TGM2 protein expression is positively correlated with the development of tumor metastasis phenotypes [14, 15]. In addition, the increased expression of TGM2 in different cancer cells can induce epithelial-mesenchymal transformation (EMT) and promote the invasion and drug resistance of cancer cells [11, 16, 17]. TGM2 expression can enable cancer cells to acquire the characteristics of stem cells, thus contributing to self-renewal and survival [18, 19]. Recent studies have shown that TGM2 protein also plays an important role in pancreatic cancer, and TGM2 promotes the growth and drug resistance of pancreatic cancer cells by influencing the ECM and stroma in the tumor microenvironment [20]. TGM2 is also associated with the development of some diseases, such as atherosclerosis and neurological diseases [21].

To explore the effects of TGM2 on the tumorigenicity of MDCK cells, TGM2-overexpressed (TGM2-OE) and knockout (TGM2-KO) MDCK cells were studied in vitro and in vivo, and the effects of TGM2 on cell proliferation and the proliferation of influenza A H1N1 virus were analyzed. To further explore the mechanism through which TGM2 regulates MDCK tumor-forming cells, we used real-time PCR to evaluate the expression of common downstream genes for the tumor-forming response signaling pathway. These studies contribute to a more comprehensive understanding of the potential tumor-forming molecular mechanism of MDCK cells and the biological functions of TGM2 and also provide a basis for the construction of safer and higher-yield genetically engineered cell lines.

## 2 Material and methods

Our research complies with all relevant ethical regulations of the Northwest Minzu University. The animal study protocol was approved by the Experimental Animal Ethics Committee of Northwest Minzu University (protocol code xbmu-sm-2022022, 7 Mar. 2022). for studies involving animals. Our study with observational experimental design was carried out in compliance with the ARRIVE guidelines. Anesthesia method: Inhalation anesthesia. Anesthetic name: Isoflurane. Method of execution: Cervical dislocation and death.

### 2.1 Cell culture and virus

High tumorigenic MDCK cells were stored in a library established for this experiment and were purchased from American Type Culture Collection (ATCC, USA). Low tumorigenic MDCK cells were domesticated and preserved in our laboratory. MDCK adherent cells (MDCKADH and CCL-34 (ATCC) were cultured in Dulbecco’s Modified Eagle Medium (DMEM) containing 10% fetal bovine serum (CellMax, SA311.01) (Lanzhou Bailing Biotechnology Co., Ltd.) in T25 cell bottles (Corning, 430641) at 5% CO_2_ and 37°C. TGM2-OE lentivirus and TGM2-OE negative control lentivirus (Con) (virus titer: 1×10^9^TU/mL) were purchased from Shanghai Jikai Biotechnology Co., Ltd. Lentivirus packaging vectors psPAX2, PCMV-VSV-G, and lenti-CRISPRv2 knockout vectors were stored in our laboratory. The H1N1 influenza virus strain number was X-275. The strain was provided by Wuhan Institute of Biological Products Co., Ltd. (Wuhan 430000; Zhangjiayou).

### 2.2 Chemical reagents

TGM2 monoclonal antibody D11A6 (Cell Signaling Technology), Rabbit antibody β-Actin ab6276 (Abcam), Horseradish peroxidase-labeled goat anti rabbit IgG monoclonal antibody 7074P2 (Cell Signaling Technology), Puromycin P8230 (Solabao Biotechnology Co., Ltd), fibronectin (FN) F8180 (Solabao Biotechnology Co., Ltd), Matrix adhesive 356234 (Corning), CCK8 reagent C0037 (Biyuntian Biotechnology Company), Transwell cell CON-3422 (Corning), 1% chicken erythrocytes HQ80071 (Hongquan Biology), reverse transcription kits (Solabao), and real-time PCR (QRT PCR) kits AG11705 (Solabao) were used.

### 2.3 Construction of TGM2 overexpression stable cell line and screening of stable cell lines

MDCK cells were evenly inoculated into 12-well plates. When the cell density reached 50%-70% the next day, TGM2-OE plasmid and negative control plasmid were transfected into the cells through lentiviruses. After 12 hours, the culture medium was changed to DMEM containing 10% fetal bovine serum. After incubation in a 5% CO_2_ and 37°C incubator for 48 hours, the fluorescence expression was observed under a fluorescence microscope, and the cells were subcultured. After the cells adhered to the wall, puromycin with a concentration of 4 μg/mL was added to screen cellular resistance. After continuous screening for 15 generations, a stable cell line with TGM2 overexpression was obtained.

### 2.4 Construction of knockout vector

#### 2.4.1 Preparation of electrotransfer target cells and monoclonal cell lines

An MDCK cell suspension (3 × 10^6^) was placed in a sterile tube and centrifuged at 1000 g for 5 minutes. The supernatant was discarded, and the cell precipitate was resuspended with 600 μL buffer R. Afterward, 30 μg CRISPR-UTM expression plasmid without endotoxins was added, and the cells were mixed evenly. BuffereE2 (3 mL) was added to an electric shock cup and placed in the card slot of the electromotor. A 100 μL electric rotation pipette tip was used to absorb the mixture of cells and plasmids. The mixture was transferred to the electric shock cup, the electric rotation conditions were set, and the electric shock procedure was initiated. After electroporation, the cells were inoculated into six-well plates for further culture. After 24–48 hours of transfection, the electrotransformation effect was observed under a microscope. The hole with the best cell viability and green fluorescent cell percentage was selected and replaced with culture medium containing purifosfomycin. The cells were screened for 2–3 days, digested into a single-cell suspension after screening, and counted.

#### 2.4.2 Screening and sequencing identification of monoclonal antibodies against TGM2 knockout cell lines

After the cells were inoculated into 96-well plates and cultured for one week, the growth of clones was observed, and the clones were labeled. Monoclonal identification was performed when the cell density reached 40% - 60%. The culture medium in the 96-well plate was poured out, 100 μL ubigene rapid nucleic acid release reagent was added, and the cells were left at room temperature for 10 min. After centrifuging at 3000 rpm for 5 min, the nucleic acid in the cell was released into the supernatant, and the knockout region was amplified by PCR.

### 2.5 Cell proliferation experiment

The effects of TGM2 overexpression and knockout on the proliferation activity of MDCK cells were measured by trypsin digestion. Cell suspensions of TGM2-OE, Con, TGM2-KO, and WT MDCK cells in good growth condition were prepared by trypsin digestion. The cell concentration was adjusted to 4×10^3^ cells/mL. The cells were inoculated on 24-well cell culture plates, with 0.5 mL per well, and cultured in a 5% CO_2_ and 37°C incubator. The solution was changed every 48 h. The cells were digested with trypsin and counted every 24 h. Three replicates were performed in parallel in each group for 10 days. The average values were calculated, and cell growth curves were created.

### 2.6 Cell migration experiment

MDCK cells in the logarithmic growth stage were digested with trypsin. After counting, the cells were inoculated into each well of a six-well plate, with 3 × 10^5^ cells in each well. After the cells were confluent, 200 μL of sterilized cells was used for the experiment, and a 200 μL pipette tip was used to scratch the central area of the cell monolayer in a straight line. The scratched cells were washed with PBS, and the scratch width was photographed with an inverted microscope at 0 h and measured with Image J software. The cells were cultured in a constant temperature incubator at 5% CO_2_ and 37°C for 12, 24, 36, and 48 h. After washing with PBS, the cells were observed under an inverted microscope. Pictures and videos were taken at the same positions, and the scratch width was measured with Image J software.

### 2.7 Cell invasion experiment

Before the experiment, 200 μL serum-free DMEM medium containing 10 g/L BSA was added to the upper chamber and incubated at 37°C for 30 min to hydrate the basement membrane. The cells in each group were starved overnight with serum-free medium, digested with trypsin and resuspended. Afterward, the cells were centrifuged at 1000 rpm for 5 min, and the culture medium was discarded. The cells were washed 1–2 times with PBS, resuspended with serum-free DMEM medium containing BSA, and placed in single-cell suspensions with 2.5 × 10^5^/mL. The suspensions were added to the upper chamber along the side wall to avoid bubbles, gently mixed, and inoculated into Transwell chambers coated with Matrigel. Subsequently, 600 μL of 20% serum DMEM was added to the lower chamber of the chamber, and the cells were cultured at 37°C with 5% CO_2_ for 24 h. After removing the Transwell chamber, the cells that migrated to the lower chamber were analyzed. The chamber was gently turned upside down on absorbent paper to remove the culture medium and rinsed with PBS twice. The cells in the upper chamber were wiped off the membrane with a cotton swab. The Transwell lower chamber was fixed in 4% paraformaldehyde for 30 min, washed twice with PBS, dried at room temperature for 5 min, stained with 0.1% crystal violet for 30 min, rinsed twice with PBS and dried at room temperature. The number of invading cells was calculated from three randomly selected visual fields under an inverted microscope.

### 2.8 Tumor formation experiment in nude mice

Four to six weeks old BALB/c female nude mice were divided into six groups. Cervical cancer cells (HeLa) were used as a positive control group, human embryonic lung fibroblasts (MRC-5) with no-tumorigenicity cells were used as a negative control group,TGM2-OE group compared with Con group, TGM2-KO group compared with Wild Type MDCK CCL-34 P60(WT) group, with 10 mice in each group, sixty mice in total. The cells in each group were digested with trypsin and centrifuged at 1000 rpm/ min for 5 minutes. The supernatant was discarded to make 5 × 10^6^/mL and 5 ×10^7^/mL cell suspensions, which were subcutaneously injected into the back of the nude mice. Each mouse was inoculated with a cell suspension of 0.2 mL. HeLa cells were used as the positive control to determine whether the model was successful. After inoculation, the nude mice were raised in SPF animal rooms. The mice were observed twice a week. During this period, the weight was recorded, and the tumor volume was determined (tumor length (a) and width (b) were measured with vernier caliper, and tumor volume = (a) × b2 / 2). The weekly tumor volume growth map, final tumor volume, and final body weight map of nude mice were recorded.

### 2.9 IAV infection and virus titer detection

TGM2-OE, Con, TGM2-KO, and WT cells were evenly plated in 12-well plates. After the cells were confluent, they were washed once with PBS to remove the residual serum. Afterward, H1N1 influenza A virus containing 4 μg/mL TPCK trypsin (MOI = 0.01) was used to infect the two groups of cells, and the cells were placed in a 37°C and 5% CO_2_ incubator for 1 h for adsorption. After 1 h, the medium was replaced with serum-free medium containing 4 μg/mL TPCK trypsin, and the cell culture supernatants were collected after 48 h. Finally, the TCID_50_ for the virus titer was determined. After statistical analysis, the virus titer (TCID_50_) was calculated using the Reed-Muench formula.

### 2.10 Real-time PCR

The total RNA of TGM2-OE, Con, TGM2-KO, and WT cells in good growth condition was extracted, and the RNA concentration was measured with an enzyme labeling instrument (Thermo company). Afterward, 1 g of the total RNA was reverse-transcribed using the cDNA as the template. The real-time fluorescence quantitative reaction conditions were in accordance with the instructions for the fluorescence quantitative PCR reverse transcription kit and the all-in-one qPCR mix kit. The reaction conditions were: pre denaturation at 95°C for 30 s, denaturation at 95°C for 5 s, and annealing at 60°C for 35 s, for a total of 40 cycles. RT qPCR reaction was carried out with the Applied Biosystems 7500 fluorescence quantitative PCR instrument, and the values obtained were standardized with GAPDH as the internal reference. The results of quantitative PCR were analyzed using the 2-ΔΔCT method.

### 2.11 Western blotting

The expression of TGM2 protein was detected in TGM2-OE, Con, TGM2-KO, and WT cells. The expression levels of NP and NS1 proteins were detected after infecting the above cells with H1N1 influenza A virus. The process is summarized as follows: when the cells in the T25 cell culture bottle were covered with a single layer, cell samples from each group were washed with PBS three to five times. The cells were collected with a cell scraper, and 500μl radioimmunoprecipitation (RIPA) buffer was added to the cell culture pyrolysis fluid (1% phenylmethanesulfonyl fluoride was added to the lysate). The cells were lysed on ice for 30 minutes and centrifuged at 4°C and 12000 rpm for 10 minutes. The supernatant was removed, and the protein concentration was determined with the bicinchoninic acid (BCA) assay. The protein concentration of each sample was adjusted to 1.5μg/μL for SDS-PAGE gel electrophoresis. The protein was transferred to polyvinylidene difluoride (PVDF) membranes (Millipore, Bedford, MA) with the wet transfer method. Tris-buffered saline with polysorbate 20 (TBST) solution and 5% skimmed milk were added, and the membranes were sealed at room temperature for 2 h. TGM2 primary antibody (1:1000, Cell Signaling) and β-Actin primary antibody (1:5000, Abcam) were added and incubated overnight at 4°C. The cells were washed three times with TBST for 5 minutes each time. The corresponding secondary antibodies (1:10000, Cell Signaling) were diluted with blocking solution and incubated for 1 h at room temperature. The membrane was washed with TBST for 5 min each time and with TBS once for about 10 min. Finally, the results were analyzed using ECL (PerkinElmer Inc, MA) and Tanon 5500 gel imaging (Tanon Science Technology Co., Ltd Shanghai, China) systems.

### 2.12 Data analysis

The experimental data are expressed as "mean ± standard deviation". Graphpad Prism 8.0 was used for one way ANOVA. Values with P < 0.05 and P < 0.01 were considered significant, and those with P < 0.001 were extremely significant.

## 3 Results

### 3.1 TGM2 expression was significantly up-regulated in MDCK low tumor-forming cells

**Fig 1:**
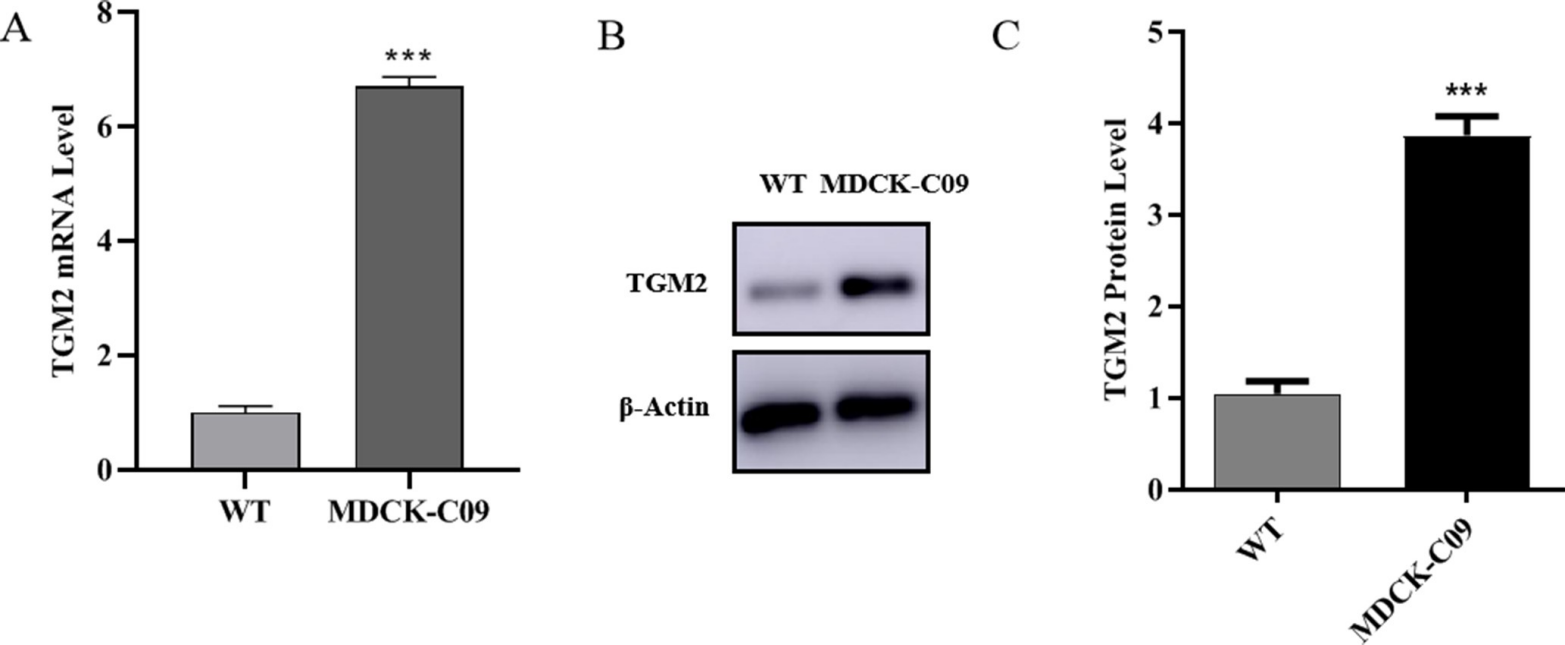
Expression of TGM2 in high and low tumor-forming MDCK cells detected by real-time PCR and Western blotting. (A). TGM2 mRNA levels in high and low tumorigenesis MDCK cells determined by real-time PCR. (B). TGM2 protein levels in high and low tumor-forming MDCK cells were determined by Western blotting and densitometrically quantified and normalized to β‐actin (C).

In our previous work, we screened a low tumorigenic cell line (MDCK-C09) and a high tumorigenic cell line [22] (WT) through monoclonal experiments. Studies have shown that TGM2 is associated with cell tumorigenesis. Therefore, in order to explore whether TGM2 affects MDCK cell tumorigenesis, we first assessed whether TGM2 expression differs in high and low tumorigenic cells. Quantitative RT-PCR and Western blotting were used to measure the mRNA and protein levels of the TGM2 gene, respectively. The PCR results showed (Fig 1A) that the expression level of TGM2 mRNA was significantly higher in MDCK-C09 cells compared with the level in high tumor-forming cells. Western blotting results showed (Fig 1B and 1C) that the TGM2 protein level was significantly up-regulated in low-oncogenic MDCK cells, and the protein levels differences were consistent with the pattern of mRNA level differences. This suggests that TGM2 may be negatively correlated with tumorigenesis in MDCK cells. The experimental data were expressed as "mean ± standard deviation" and P < 0.001 indicated extremely significant differences.

### 3.2 TGM2 overexpression inhibits the proliferation, migration, and invasion of MDCK cells

**Fig 2:**
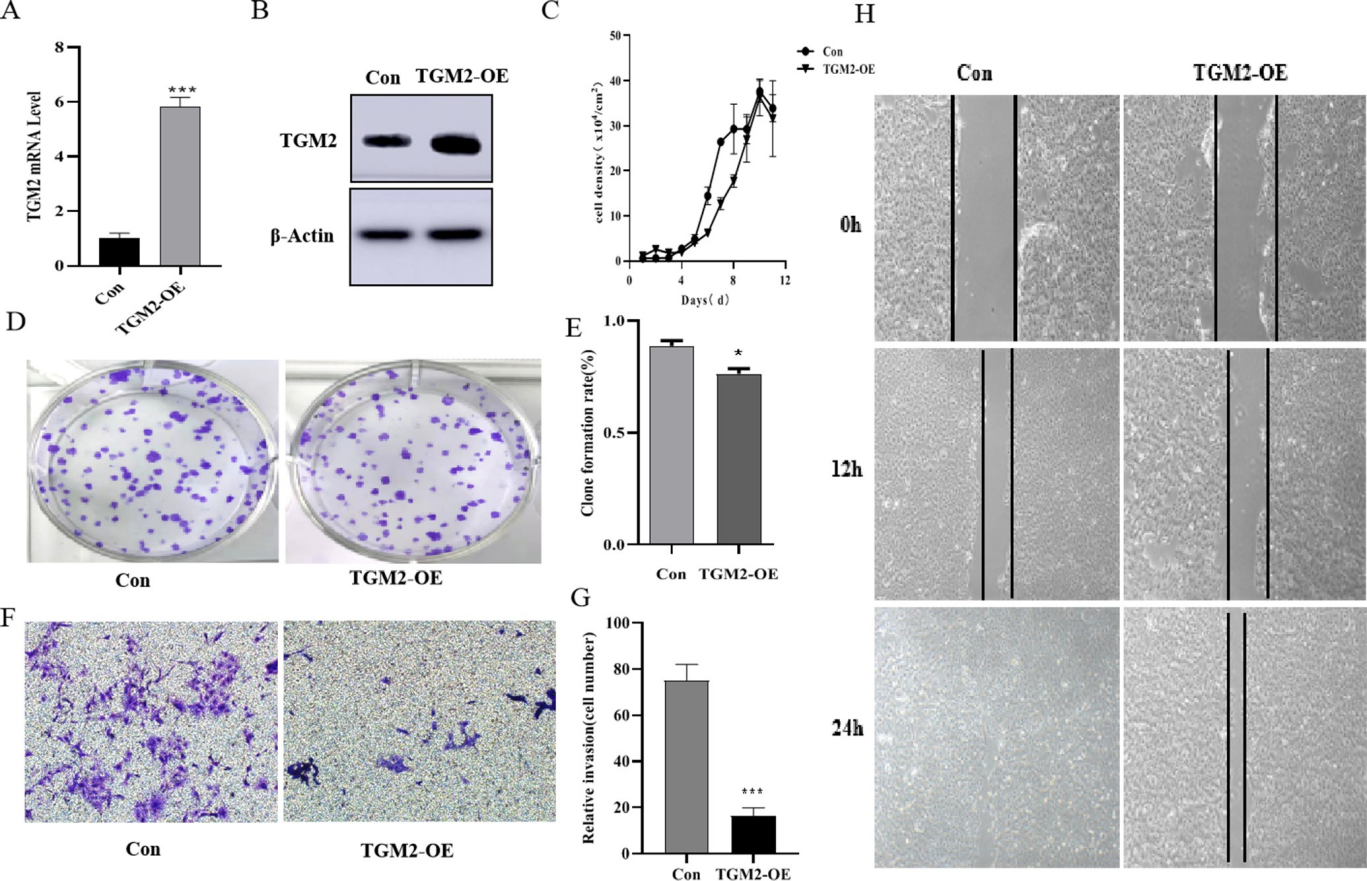
Effects of TGM2 overexpression on proliferation, migration, and invasion of MDCK cells. (A). mRNA levels of TGM2-overexpressed cells were determined with real-time PCR. (B). Protein levels of TGM2-overexpressed cells determined by Western blotting. (C). Effects of TGM2 overexpression on proliferation ability of MDCK cells determined by cell counting; (D,E) Effects of TGM2 overexpression on plate clone rate of MDCK cells determined by plate clone formation assays. (F,G) The effects of TGM2 overexpression on the invasion ability of MDCK cells were determined using Transwell assays. (H) The effects of TGM2 overexpression on the migration ability of MDCK cells were determined by scratch tests. TGM2-overexpressed cells (TGM2-OE); Vector control cell (Con). The experimental data are expressed as "mean ± standard deviation". *P < 0.05 and **P < 0.01 indicate significant differences, and ***P < 0.001 indicates extremely significant differences.

To explore the effects of TGM2 on the tumorigenesis of MDCK cells, we first constructed TGM2-OE MDCK cell lines using a lentiviral vector-mediated expression system. Real-time PCR and Western blotting results showed (Fig 2A and 2B) that the protein expression level and mRNA level of TGM2 in the TGM2-OE cell lines were significantly up-regulated compared with the levels in the vector control group. In vitro tumorigenicity analysis is mainly focused on changes in cell proliferation, migration, and invasion functions. The effects of TGM2 gene overexpression on the proliferation of MDCK cells were first investigated. The cell count results are shown in Fig 2C. Over time, the proliferation activity of the overexpressed cells was lower than that of the vector control cells. The results of the plate clone formation experiment are shown in Fig 2D and 2E. Compared with the vector control group, the proliferation activity and clone formation rate of TGM2-OE cells were significantly lower. Next, we measured the effects of TGM2 overexpression on the migration and invasion ability of MDCK cells. The scratch results are shown in Fig 2H. Compared with the vector control group, the scratch healing ability of cells in the TGM2 overexpression group was significantly lower. Cells in the control group were mostly healed at 24 h, unlike cells in the TGM2 overexpression group. The results of cell invasion experiments showed that (Fig 2F and 2G) compared with the vector control cells, the invasion ability and the number of cells invaded by TGM2-OE cells were significantly reduced. These results indicated that TGM2 overexpression significantly inhibited the proliferation, migration, and invasion of MDCK cells.

### 3.3 TGM2 gene knockout promoted the proliferation, migration, and invasion of MDCK cells

**Fig 3:**
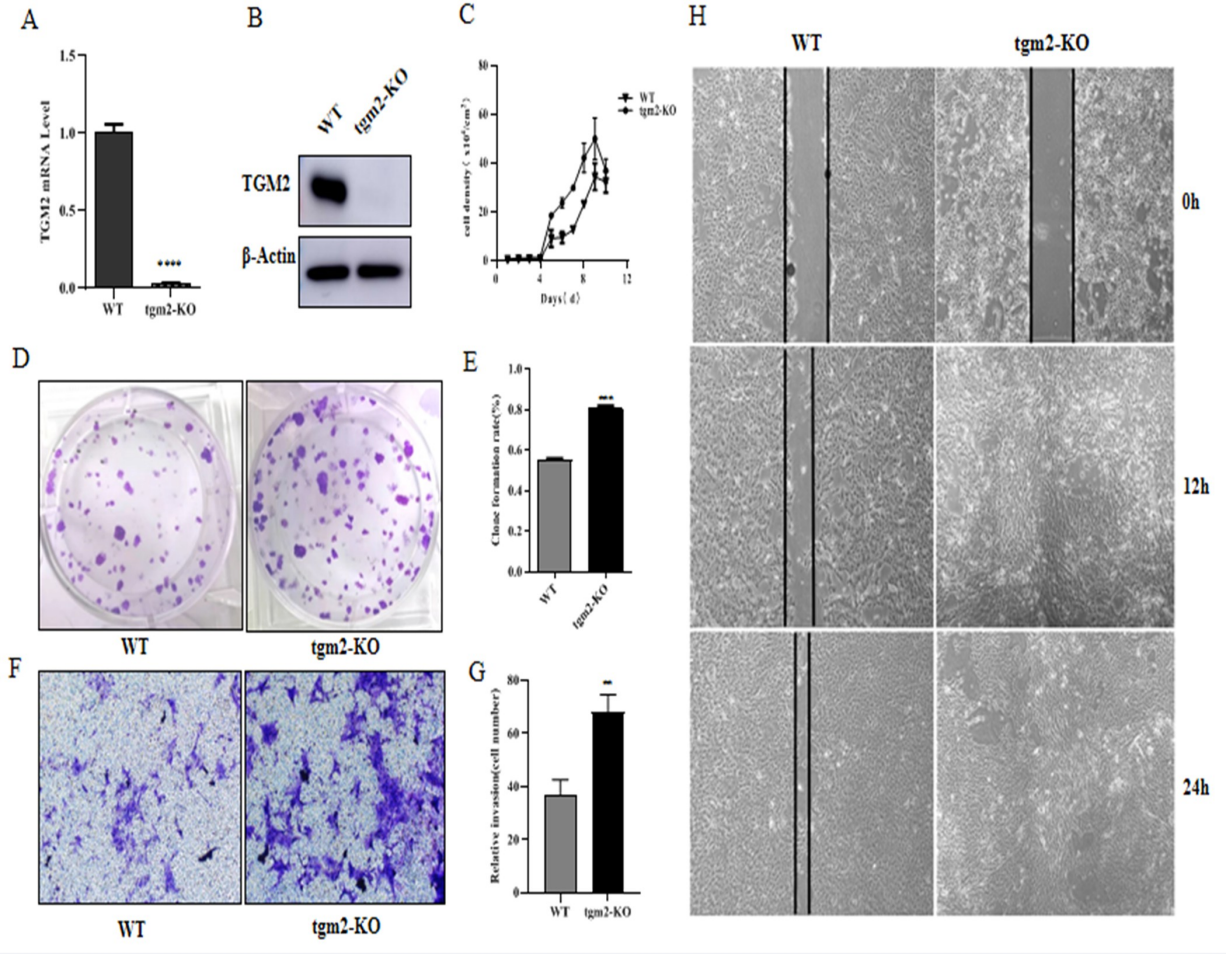
Effects of TGM2 gene knockout on proliferation, migration, and invasion of MDCK cells. (A). mRNA level of TGM2-KO cells determined by real-time PCR; (B). Protein level of TGM2-KO cells determined by Western blotting; (C). Effect of TGM2 knockout on proliferation ability of MDCK cells determined by cell counting; (D,E). Effects of TGM2 knockout on the plate clone rate of MDCK cells were determined by plate cloning experiments. (F,G). Effects of TGM2 knockout on the invasion ability of MDCK cells determined by Transwell assay; (H). Effects of TGM2 knockout on migration capacity of MDCK cells determined by scratch test. TGM2-knockout cells (TGM2-KO); Wild-type cell (WT). The experimental data are expressed as "mean ± standard deviation". *P < 0.05 and **P < 0.01 indicate significant differences, and ***P < 0.001 indicates extremely significant differences.

To further determine the role of TGM2 in regulating MDCK tumor-formation, we constructed TGM2-KO MDCK cell lines using CRISPR/CAS9 technology and evaluated the effects of TGM2 knockout using real-time PCR and Western blot (Fig 3A and 3B). TGM2 was successfully knocked out at both the mRNA and protein levels. The tumorigenicity of TGM2-KO cells was also analyzed in vitro, with a focus on the changes in cell proliferation, migration, and invasion. The cell growth curve showed that (Fig 3C) TGM2-KO cells had significantly higher proliferation activity than wild-type (WT) cells over time. Plate clone formation experiments (Fig 3D and 3E) revealed that the cell proliferation ability and clone formation rate of the TGM2-KO group were significantly enhanced, and the difference was significant compared with those of the WT group. The effects of TGM2 knockout on the migration and invasion of MDCK cells were further analyzed with scratch healing and Transwell experiments. The cell migration experiments showed (Fig 3H) that the scratch healing ability of TGM2-KO MDCK cells was significantly higher than that of WT cells. TGM2-KO MDCK cells were completely healed at 12 h, while WT cells did not completely heal until 36 h. The cell invasion experiments showed that the invasion ability and number of cells in the TGM2-KO group were significantly higher than those of WT cells (Fig 3F and 3G). These results indicated that TGM2 knockout significantly enhanced the proliferation, migration, and invasion abilities of MDCK cells. Combined with the TGM2 overexpression results, these findings confirmed that TGM2 could inhibit tumorigenesis in MDCK cells in vitro.

### 3.4 TGM2 inhibits the proliferation of influenza A virus H1N1 in MDCK cells

**Fig 4:**
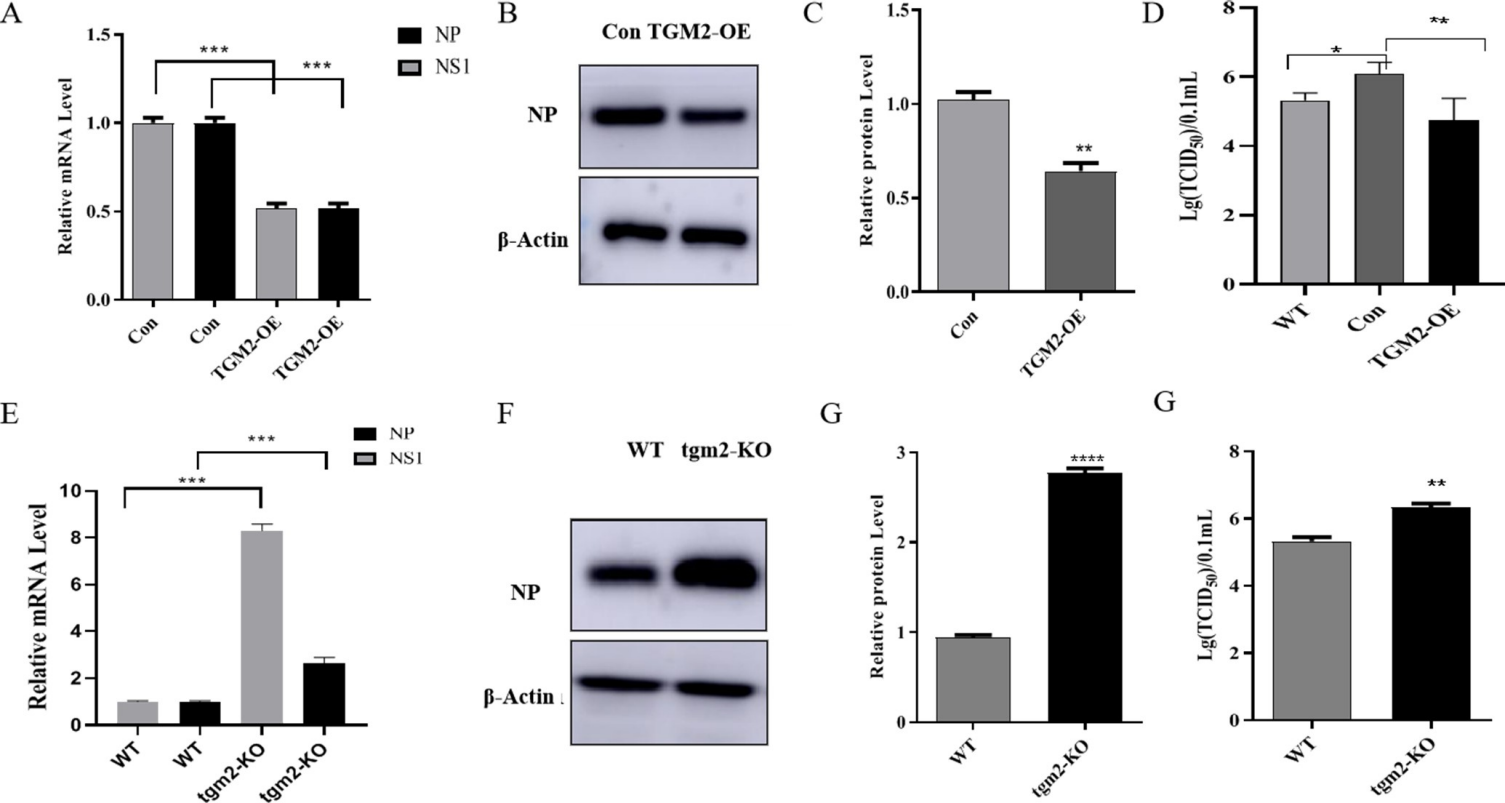
Effects of TGM2 on proliferation of influenza A (H1N1) virus. (A). mRNA levels of viral NP and NS1 genes overexpressed by TGM2 were determined by real-time PCR. (B, C). The effect of TGM2 overexpression on viral NP protein was determined by Western blotting. (D). Effects of TGM2 overexpression on titer of influenza A H1N1 virus determined by TCID_50_; (E). mRNA levels of NP and NS1 genes of TGM2 knockout virus were determined by real-time PCR. (F, G). Effect of TGM2 knockout on NP protein of the virus was determined by Western blotting. (H). Effect of TGM2 knockout on titer of influenza A (H1N1) virus measured by TCID_50_. TGM2-overexpressed cells (TGM2-OE); Vector control cells (Con); TGM2-knockout cells (TGM2-KO); Wild-type cell (WT). The experimental data are expressed as "mean ± standard deviation". *P < 0.05 and **P < 0.01 indicate significant differences, and ***P < 0.001 indicates extremely significant differences.

Since MDCK cells are the main cell line for influenza vaccine production, researchers need to pay attention not only to the problem of cell tumor formation, but also to changes in the proliferation of the virus. Some studies have shown that TGM2 is related to the proliferation of the virus. Therefore, we further explored the regulation of TGM2 on the proliferation process of the influenza A H1N1 virus. First, real-time PCR was used to measure the mRNA levels of NP and NS1 genes in TGM2-OE and TGM2-KO MDCK cells infected with the influenza A virus H1N1 at 48 h (Fig 4A and 4E). NP and NS1 genes were significantly down-regulated in TGM2-OE cells and up-regulated in TGM2-KO cells. The detection results for the viral NP protein showed that the expression of the protein was significantly down-regulated in TGM2-OE cells and up-regulated in TGM2-KO cells, which was consistent with the findings for the mRNA level (Fig 4B–4G). The median tissue culture infectious dose (TCID_50)_ was further used to detect changes in the virus titer in the supernatant of TGM2-OE and KO cells infected with the H1N1 influenza virus (Fig 4D and 4H). The titer of the H1N1 influenza virus in TGM2-OE cells was significantly lower than that in vector control cells (Con), and the virus titer in TGM2-KO cells was significantly higher than that in WT cells. Therefore, the mRNA levels of the viral genes, viral proteins, and viral titer results together indicated that TGM2 inhibited the proliferation of the influenza A (H1N1) virus in MDCK cells.

### 3.5 TGM2 inhibited the tumorigenicity of MDCK cells in nude mice

**Fig 5:**
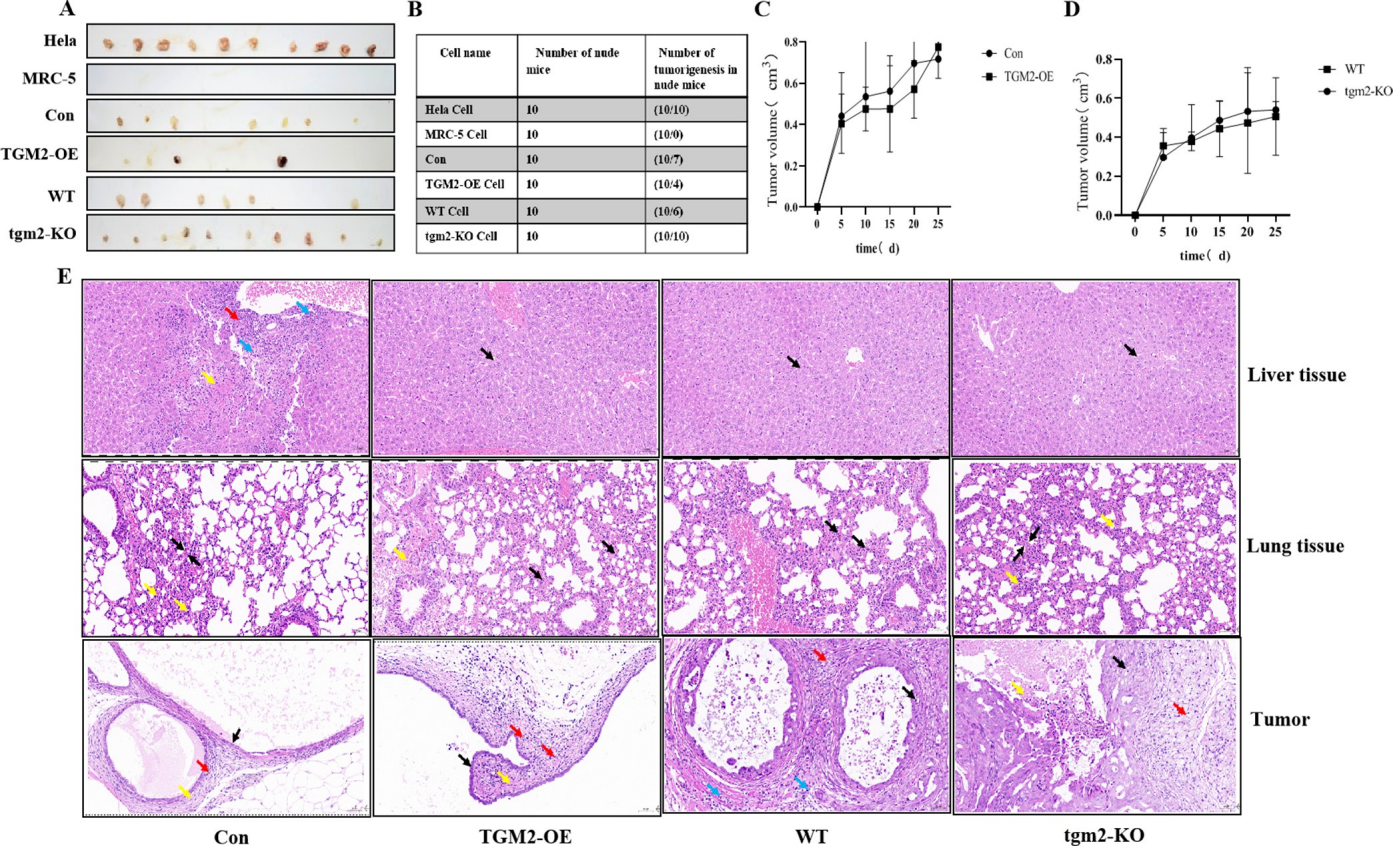
Effect of TGM2 on MDCK tumorigenicity. (A). Subcutaneous tumor display in nude mice. (B) Statistical results of subcutaneous tumor number in nude mice. (C). Effect of TGM2 overexpression on tumor volume. (D). Effect of TGM2 knockout on tumor volume. (E). Histological observation of liver, lung and subcutaneous tumors in nude mice 30 days after Con, TGM2-OE, WT, and TGM2-KO cells were injected subcutaneously (H&E staining). A large number of hepatic cells were seen in the tissues with mild watery degeneration, cell swelling, loose cytoplasm, and light staining (black arrow). Focal necrosis of the liver cells was observed with nuclear fragmentation or dissolution (yellow arrows), with a small amount of connective tissue hyperplasia (red arrows), and a small amount of lymphocyte and neutrophil infiltration (blue arrows). Positive control (HeLa cells), Negative control (MRC-5 cells), TGM2 overexpression (TGM2-OE), Carrier control (Con), TGM2 knockout (TGM2-KO), Wild type (WT).

In in vitro experiments, we found that TGM2 inhibited cell proliferation, migration, and invasion in MDCK cells. To further confirm the effect of TGM2 on tumorigenesis in MDCK cells, we conducted tumorigenesis experiments in nude mice. After the adherent cells were digested, HeLa cells were subcutaneously injected into the back of nude mice at a density of 5×10^6^ cells/mL, and MRC-5 cells were injected at a density of 5×10^7^ cells/mL, each with a cell suspension of 0.2 mL. After inoculation, the mice were fed and housed for 1 month in a specific-pathogen-free (SPF) environment (Fig 5A). All 10 nude mice injected with MRC-5 cells were tumorless, while none of the 10 mice injected with HeLa cells were tumorless. The number of subcutaneous tumors in the group injected with TGM2-OE MDCK cells was significantly less than that in the vector control group. The number of subcutaneous tumors in the TGM2-KO group was significantly higher than that in the WT group. The results are consistent with those of tumorigenesis analysis in vitro. Changes in the tumor volume (tumor length and width) were measured with vernier calipers every 5 days (Fig 5C and 5D). The number of tumors (Fig 5B) and the tumor volume in the TGM2-OE group were significantly lower than those in the vector control group. In contrast, the tumor volume and number of tumors in the TGM2-KO group were significantly higher than those in the WT group. In order to further analyze the characteristics of the tumors and the occurrence of tumor metastasis, sectioning and hematoxylin and eosin (H&E) staining were performed on the tumor, lung, and liver tissues. The results are shown in Fig 5E.

Con cells: A large number of liver cells were found in the liver tissue of nude mice, with mild watery degeneration, cell swelling, loose cytoplasm, and light staining, and obvious degeneration of liver cells was observed at the edge of local tissues. Focal necrosis of the liver cells with nuclear fragmentation or dissolution was also observed, with a small amount of connective tissue hyperplasia and a small amount of lymphocyte and neutrophil infiltration. Local thickening of the alveolar walls was found in the lung tissue with punctate infiltration of lymphocytes and neutrophils. The tumor cells were epithelioid with a high nucleo-cytoplasmic ratio and uniform nuclei. No signs of mitosis were observed. Surrounding connective tissue hyperplasia and a small amount of lymphocyte infiltration were noted.

TGM2-OE cells: An increased amount of liver tissue with liver cell degeneration was visible, with mild water cell swelling and loose light dye in the cytoplasm. Increased amounts of diffuse alveolar walls with greater lymphocyte and neutrophil infiltration were observed along with local visible bleeding. The tumor cells were epithelioid with high nuclear/cytoplasmic ratios, and the size of the nucleus was consistent. Mitosis was apparent, along with connective tissue proliferation and scattered lymphocyte and neutrophil infiltration.

WT cells: Liver cell degeneration, mild water cell swelling, and loose light dye in the cytoplasm were observed. A small amount of lymphocyte and neutrophil infiltration of the tumor cells was found in the lung alveolar wall. The gland duct to nuclear mass ratio was high, the nucleus shape was consistent, and nuclear division was apparent. Some tumor cells presented epithelioid arrangements, high nucleo-cytoplasmic ratios, uniform size and nuclei shape, less mitosis, more connective tissue hyperplasia, and rare punctate infiltration of lymphocytes and neutrophils.

TGM2-KO cells: Many liver cells with mild hydroid degeneration, cell swelling, and loose and lightly stained cytoplasm were observed. The pulmonary tissue had diffuse infiltration of lymphocytes and neutrophils and local thickening of the alveolar walls. The tumor cells had a glandular tubular shape, with high nucleo-cytoplasmic ratios and various nuclei with different shapes and sizes. Mitosis was easily apparent. Focal tumor cell necrosis and nuclear fragmentation or dissolution could be seen locally. There was more infiltration of macrophages at the tissue margins.

These results demonstrated that TGM2 inhibited the tumor formation of MDCK cells in nude mice. The tissues evaluated were relatively intact, indicating that no MDCK cell tumor metastasis occurred in the liver and lungs 30 days after the injection of MDCK cells.

### 3.6 Functional recovery experiments further demonstrated that TGM2 inhibited MDCK cell tumorigenesis

**Fig 6:**
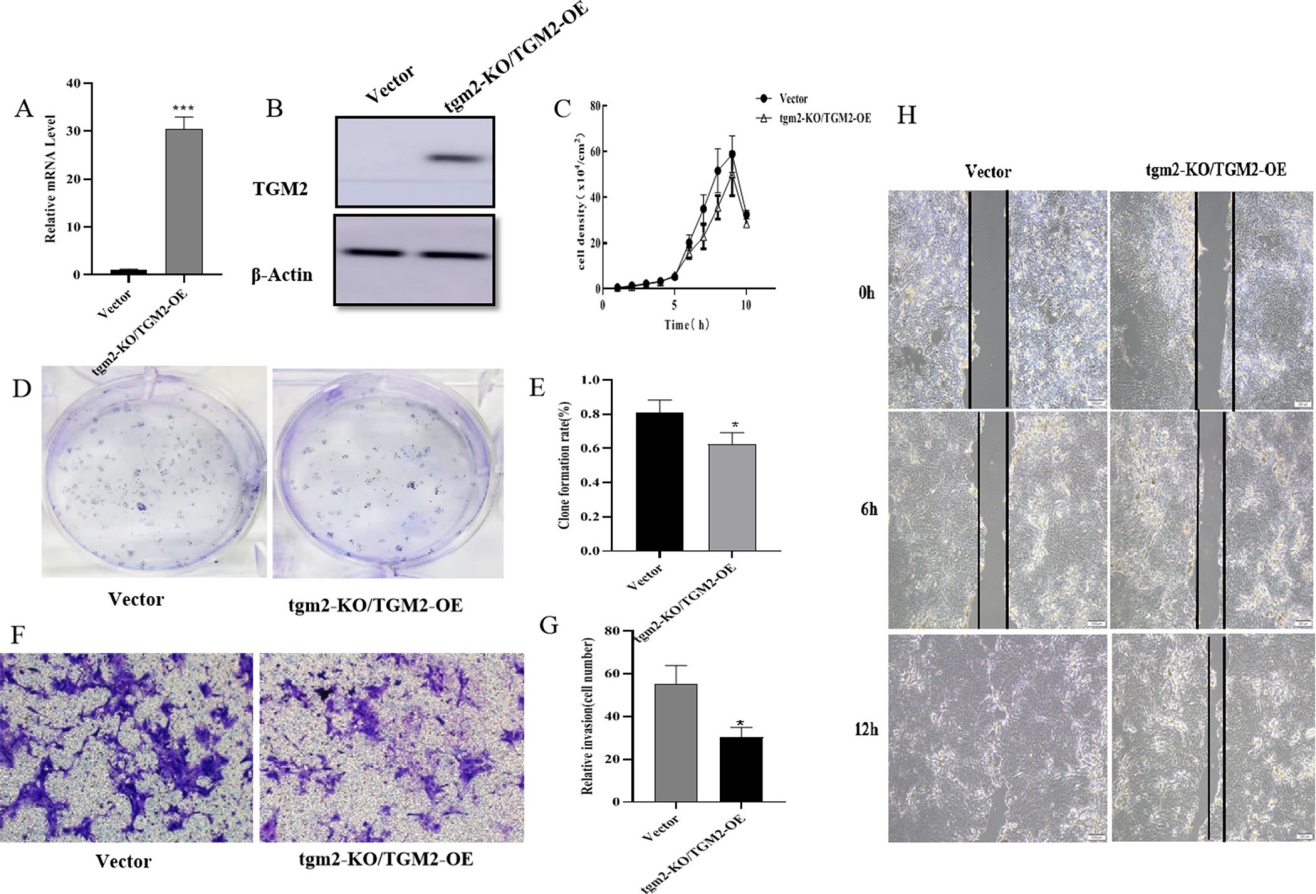
Effects of TGM2 on tumorigenicity of MDCK cells in vitro were further analyzed with functional recovery assays. (A) The TGM2 mRNA level was determined by real-time PCR in functionally recovered cells; (B) TGM2 protein level in functional recovery cells was determined by Western blotting; (C) Cell counting was used to measure the proliferation of functional recovery TGM2 cells. (D, E) Plate clone formation assay to determine the plate clone rate of functional regenerative cells; (F, G) Transwell assay was used to determine the invasion ability of functional recovery cells. (H) Changes in the migration ability of functional recovery TGM2 cells were determined with a scratch assay; TGM2 return vector control cells (Vector); TGM2-functional regenerative cells (TGM2-KO/TGM2-OE). The experimental data are expressed as "mean ± standard deviation". *P < 0.05 and **P < 0.01 indicate significant differences, and ***P < 0.001 indicates extremely significant differences.

To further confirm the function of TGM2 in MDCK cells, TGM2 knockout was performed with CRISPR/Cas9, which has a risk of non-specific gene knockout. We transferred TGM2-OE plasmids into TGM2-KO cells for functional recovery experiments. Firstly, the mRNA and protein levels of TGM2 in the functional recovery cells (TGM2-KO/TGM2-OE) were detected with real-time PCR and Western blotting, as shown in Fig 6A and 6B. The expression levels of TGM2 mRNA and protein were significantly up-regulated in TGM2-KO/TGM2-OE cells compared with the levels in vector cells. The cell growth curve showed that the proliferation ability of TGM2-KO/TGM2-OE cells was significantly lower than that of vector cells (Fig 6C). Plate cloning experiments showed that the proliferation activity and plate cloning rate of TGM2-KO/TGM2-OE cells were significantly lower than those of vector cells (Fig 6D and 6E). The effects of TGM2 recovery on the migration and invasion of MDCK cells were further analyzed with scratch healing and Transwell experiments. Cell migration results showed that the scratch healing ability of TGM2-KO/TGM2-OE cells was significantly lower than that of the vector control cells (Fig 6H). Cell invasion experiments showed that the invasion ability and number of cells in the TGM2-KO/TGM2-OE group were significantly lower than those of the vector group (Fig 6F and 6G). Combined with the above results, these findings confirmed that the functional recovery of TGM2 significantly inhibited the proliferation, migration, and invasion of MDCK cells.

### 3.7 TGM2 may affect MDCK tumorigenicity by regulating TWIST1 expression

**Fig 7:**
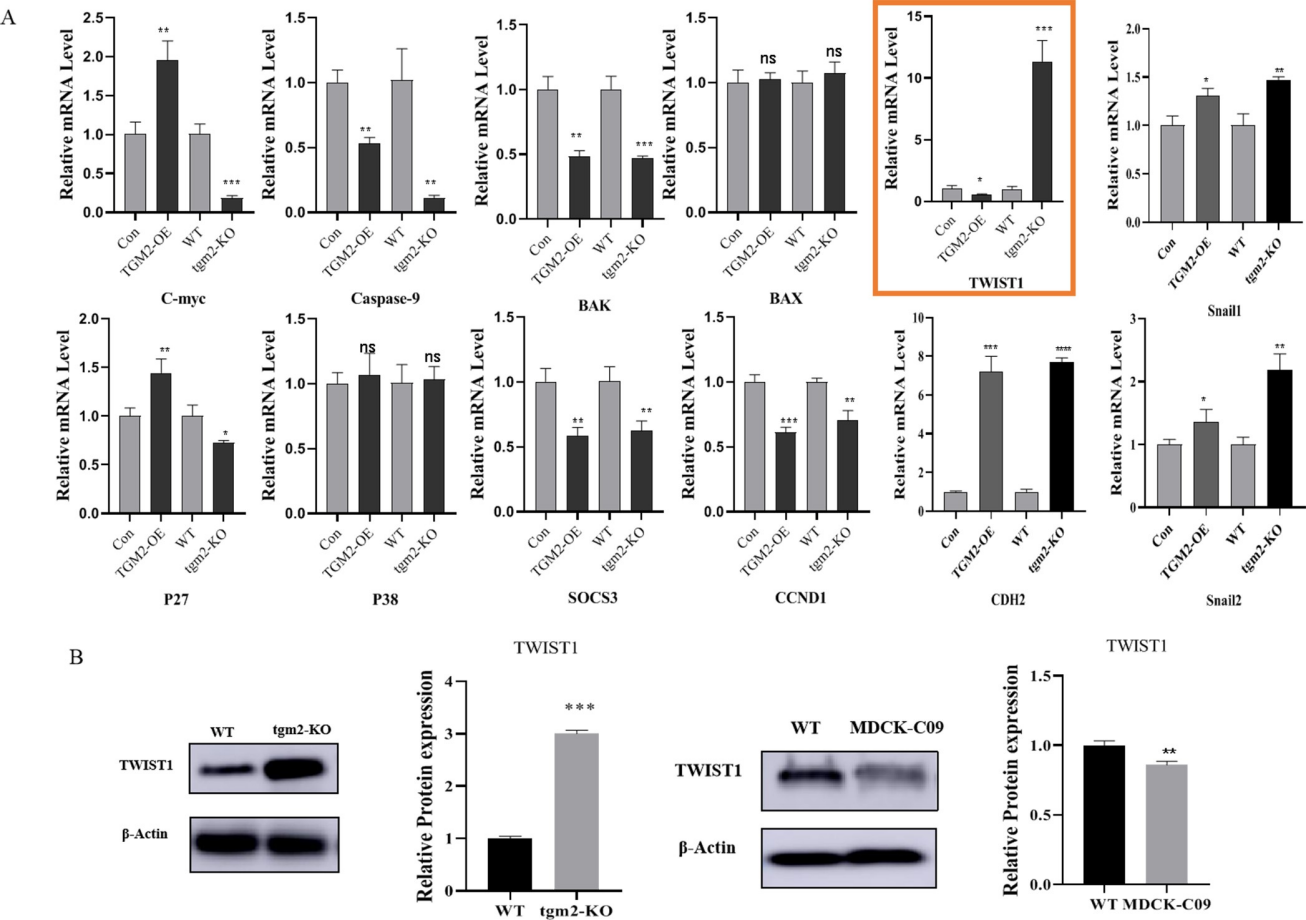
Screening of tumorigenesis-related target genes regulated by TGM2. A: Real-time PCR showing the C-myc, Caspase 9, BAK, BAX, TWIST1, CDH2, P27, P38, Snail1, SOCS3, Snail2, and CCND1 mRNA levels. B: TWIST1 protein levels in high and low tumor-forming MDCK cells and TGM2-KO cells were determined by Western blotting and densitometrically quantified and normalized to β‐actin. The experimental data are expressed as "mean ± standard deviation". *P < 0.05 and **P < 0.01 indicate significant differences, and ***P < 0.001 indicates extremely significant differences.

To preliminarily explore how TGM2 affects MDCK cell tumorigenesis, we focused on the classical signaling pathways reported to be related to cell tumorigenesis, including the MAPK, mTOR, PI3K-Akt, and WNT signaling pathways, and used real-time PCR to detect the expression changes for the downstream genes of these signaling pathways after TGM2 overexpression and knockout. C-myc, Caspase 9, BAK, BAX, TWIST1, CDH2, P27, P38, Snail1, SOCS3, Snail2, CCND1 (Fig 7A), and TWIST1 were significantly up-regulated in TGM2-KO cells and down-regulated in TGM2-OE cells. Protein analysis results were consistent with the trend of mRNA changes, the expression of TWIST1 was significantly up-regulated in TGM2 knockout cells. The protein level of TWIST1 was significantly reduced in MDCK low tumorigenesis cells, suggesting a positive correlation between TWIST1 and MDCK tumorigenicity (Fig 7B). Studies have shown that TWIST1 protein, an important transcription factor in the process of EMT transformation, plays an important role in the migration and invasion of cancer cells and can positively regulate the migration and invasion of cancer cells [23].

## 4 Discussion

TGM2 protein is mainly located in the cytoplasm of the cell, and part of the protein can be secreted outside the cell for corresponding physiological functions at different locations in the cell [24]. Recent studies have shown that TGM2 plays an important role in various physiological processes such as cell growth and differentiation, stabilization of the ECM, and remodeling of the cytoskeleton [23]. Studies have shown that TGM2 is related to the proliferation of different cells. For example, ear mesenchymal stem cells (EMSCs) isolated and cultured in vitro can secrete a variety of neurotrophic factors, including nerve growth factor, neurotrophin-3, and brain-derived neurotrophic factor, which play important roles in the proliferation and differentiation of nerve cells [25, 26]. However, TGM2 overexpression promotes the proliferation and differentiation of EMSCs by promoting ECM deposition and the cross-linking of endogenous neural factors. In addition, TGM2 gene knockout inhibits the proliferation of glioma cells and reduces the expression of inhibitor of differentiation 1 (ID1), the downstream medium through which TGM2 regulates the proliferation of glioma cells. TGM2 can regulate the proliferation of glioma cells by regulating the expression of ID1 through the PI3K/AKt pathway.

In addition to regulating cell proliferation, TGM2 plays an important role in the migration and invasion of tumors; for example, the expression level of TGM2 is increased in breast, colon, and ovarian cancers [27], and the protein is up-regulated in metastatic lung cancer cells [28]. Other studies have shown that epigenetic silencing of TGM2 occurs in primary breast tumors, including invasive tumors and ductal carcinoma in situ (DCIS). TGM2 provides phenotypic advantages to cancer cells by cross-linking ECM proteins such as fibronectin, laminin, and collagen [29]. In cancer cells, TGM2 protein is associated with β1, β3, and β5 integrin, and TGM2 expression is positively correlated with the process of cell adhesion, migration, and invasion of the ECM [30]. Recently, TGM2 was reported to affect tumor cell EMT transformation by promoting protein cross-linking and activating stromal fibroblasts [20]. TGM2 has been shown to induce transcriptional regulation by recruiting Snail promoter sequences [31]. In the process of EMT transformation, up-regulated expression of transcription factors, such as Snail1, Twist1, Zeb1, and Zeb2, can increase the migration and invasion of cancer cells. Similarly, TGF-β is considered an effective inducer of EMT under normal and pathological conditions, and up-regulated TGM2 expression can activate TGF-β and participate in EMT transformation [32]. In the above studies, TGM2 showed a positive role in regulating the migration and invasion of cancer cells and participating in EMT. Many studies have also shown that TGM2 has different functions in different cells. In more recent studies, TGM2 was found to inhibit the proliferation, migration, and invasion of cancer cells. Studies have shown that TGM2 overexpression through drug-induced treatment can reduce the proliferation, survival, and migration of melanoma cells [33, 34]. Nimesulide is a non-steroidal anti-inflammatory drug that can reduce b16-F10 cell proliferation by inducing the up-regulation of TGM2 expression [35]. Compared with normal cells, the expression of TGM2 in colon cancer cells is lower, and TGM2 is a potential growth suppressor of colon cancer in colorectal cancer cells [36]. A recent study showed that the invasion of SW480 colon cancer cells was significantly enhanced by the inhibition of TGM2 protein expression. The results of this study demonstrated that TGM2 gene knockout can significantly enhance the proliferation, migration, and invasion ability of MDCK cells in vitro and significantly increase their tumorigenesis rate in nude mice, whereas TGM2 overexpression can significantly inhibit the tumorigenesis of MDCK cells. Combined with the functional recovery experimental results after TGM2 deletion, these findings confirmed that TGM2 is involved in the negative regulation of cell proliferation in MDCK cells. However, the role of TGM2 in cancer is still controversial, with some reports suggesting it is a potential tumor suppressor [37] and others suggesting it is a tumor promoter [38]. We speculate that there may be two main reasons for the different results in these studies. First, the mechanism of cell transformation into tumors is well organized and cell-specific. The tumor development mechanism varies in different cells; for example, in B16-F10 melanoma cells, the B16-F10 melanoma cell migration, adhesion, and invasion is associated with increased quinizolin, which induces TGM2 expression [39]. Decreased proliferation of B16-F10 melanoma cells is associated with increased TGM2 overexpression, which is beneficial to melanoma formation and EMT [40]. Second, the biological functions of TGM2 may differ in different tissues and cells. The results for TGM2 in other cells from different species may differ from those in MDCK cells. More studies are needed to understand how TGM2 affects tumorigenesis; this topic will be the focus of our next study.

In this study, we screened the possible downstream target genes of TGM2 and found that the expression of Twist family bHLH transcription factor 1 (TWIST1) was significantly affected by TGM2. TWIST1 expression was significantly up-regulated by TGM2 knockout, whereas TWIST1 expression was significantly down-regulated by TGM2 overexpression. Studies have shown that TWIST1 protein is closely related to the migration and invasion of tumors. In cancer cells, TWIST1 can positively regulate the migration and invasion of cancer cells. TWIST1 promotes the invasion and metastasis of these cells as well as the phenotype of cancer stem cells, thus promoting drug resistance [41, 42]. Up-regulation of TWIST1 protein expression in breast cancer cells can promote EMT, stem cell phenotypes, and tumorigenicity [43], whereas down-regulation can inhibit the EMT process without affecting growth at the primary tumor site [44]. In human non-small cell lung cancer (NSCLC), TWIST1 knockout can inhibit tumor growth [45]. Currently, it remains unknown whether TGM2 is involved in the tumorigenesis of MDCK cells through its interaction with TWIST1 protein. The interactions between TGM2 and TWIST1 protein and the tumorigenesis mechanism of TGM2-regulated MDCK cells require further study.

In this study, we found for the first time that TGM2 inhibited the proliferation of influenza A (H1N1) virus in MDCK cells. Among the functions of TGM2, post-translational modification of proteins mediated by TGM2 is involved in viral replication. Inhibition of viral replication by TGM2 alters protein-protein interactions and prevents the formation of virus-host protein complexes through post-translational modifications of host cell proteins. Studies have shown [46] that TGM2 can indirectly regulate the replication of HIV virus, and eukaryotic translation initiation factor (EIF)-5A plays an important role in the replication of HIV. TGM2 regulates the replication of HIV by changing the activity of EIF-5A. Heat shock protein (HSP) interacts with a variety of viruses. In in vitro experiments, HSP was shown to be a substrate for TGM2 and may affect the ATP-dependent chaperone activity of HSP and the interactions between HSP and viral proteins [46]. Glyceraldehyde 3-phosphate dehydrogenase (GAPDH) can interact with influenza virus, hepatitis A virus, and hepatitis C virus, and the abundance of GAPDH in the cytoplasm as well as the instability of RNA suggests that GAPDH can directly affect viral translation and replication [47]. GAPDH is the acyl receptor of TGM2 [48]. In this experiment, TGM2 knockout promoted the proliferation of H1N1 influenza A virus in MDCK cells, while overexpression of TGM2 inhibited the proliferation of the virus. The results of this study were consistent with previous reports that TGM2 gene may be a potential antiviral gene. At present, the regulatory effect of TGM2 on influenza virus proliferation has not been reported, and the mechanism by which TGM2 regulates the replication of H1N1 influenza A virus in MDCK cells remains to be further explored.

Decades of research have shown that MDCK cells are an ideal cell matrix for influenza vaccine production compared with other available cell matrix vaccines. This is because MDCK cells have optimal influenza virus replication properties, making them the preferred cells for influenza virus isolation in World Health Organization (WHO) influenza surveillance networks and academic and diagnostic laboratories. Isolation of influenza viruses in eggs results in mutations that bind the virus to antibodies against the hemagglutinin gene. These mutations do not occur in MDCK cells. MDCK-isolated influenza viruses exhibit higher specificity and better safety. Additionally, high-dose MDCK cell lysates and cell DNA preparations do not cause cancer. For vaccine strains that change annually, especially in the case of emerging pandemic influenza viruses, the direct use of strains isolated from MDCK cells for vaccine production will significantly reduce the time required for each strain. However, due to the complexity of influenza vaccine strain selection and vaccine production, as well as the constant evolution of virus strains, various challenges remain in the production of influenza vaccines using MDCK cells. Currently, improving the titer of the influenza virus and reducing tumor-formation by MDCK cells are problems that must be addressed. In this study, we found for the first time that TGM2 had an inhibitory effect on the tumorigenicity of MDCK cells. We also found that TGM2 was involved in the regulation of MDCK cell proliferation and influenza virus proliferation. We expect to further understand the role of TGM2 protein in the tumorigenesis of MDCK cells, better understand the tumorigenesis function of TGM2 in MDCK cells, and provide a basis for the construction of high-yield genetically engineered vaccine cell lines.

## Supporting information

S1 File Animal ethics. (PDF)

S2 File Raw data of the gels and blot. (PDF)

S3 File (PDF)
