# Supplementary material for: TGM2 inhibits the proliferation, migration and tumorigenesis of MDCK cells
Source: PLoS One. 2023 Apr 28;18(4):e0285136. doi: 10.1371/journal.pone.0285136 (PMC10146566; doi:10.1371/journal.pone.0285136)
Supplement: S1 File — (PDF) [file pone.0285136.s001.pdf]

Approval of experimental Animal Ethics of Northwest Minzu University

No.: xbmu-sm-2022022 Application Date: 7 March 2022

Name of animal Research Project: Study on TGM2 regulating the tumorigenicity of MDCK cells

Project Category: The basic research

Summary of animal Experiment content: Nude mice were injected subcutaneously on the back, and each was inoculated with 0.2ml cell suspension. After inoculation, nude mice were raised in SPF animal room. Observe twice a week, record the weight of nude mice, measure the tumor volume (tumor length (a) width (b)) with vernier caliper, and the tumor volume = ($a\times b^2/2$) the weekly tumor volume growth map, final tumor volume and final body weight map of nude mice should be recorded.

Project Leader: Liu Zhenbin		Tel. +86-18919144966
Project executive: Guo Shouqing		Tel. +86-15101292196
Animal usage	Animal strain, breed, grade: （4-7w）：BALB/c nude mice	Average weight: 18 g
	Quantity: 60	Feeding time: 2021.8.14-2021.9.11
	Anesthesia method: Inhalation anesthesia	Anesthetic name: Isoflurane
	Method of execution:<br>Cervical dislocation and death	Treatment of animal residues: Put it into a garbage bag and hand it over to the school Animal Center for treatment
	Start date of animal experiment: August 14, 2021	End Date: September 11, 2021

Declaration: I will consciously accept the supervision and inspection of the Experimental Animal Ethics Committee of Northwest Minzu University, and ensure that the above materials are objective and reliable.

Signature of the project leader: Zhenbin Liu

Signature (seal) of project Executor: Shouqing Guo

Date: 7 Mar 2022

Opinion of Collage

Chief signature: Jialin Bai

Seal:

Date: 7 Mar, 2022

Opinion of ethics Committee on approval:

Chief signature: Shengdong Huo

Seal:

Date:
