# Supplementary figures and images for: TGM2 inhibits the proliferation, migration and tumorigenesis of MDCK cells
Source: PLoS One. 2023 Apr 28;18(4):e0285136. doi: 10.1371/journal.pone.0285136 (PMC10146566; doi:10.1371/journal.pone.0285136)

Fig 1

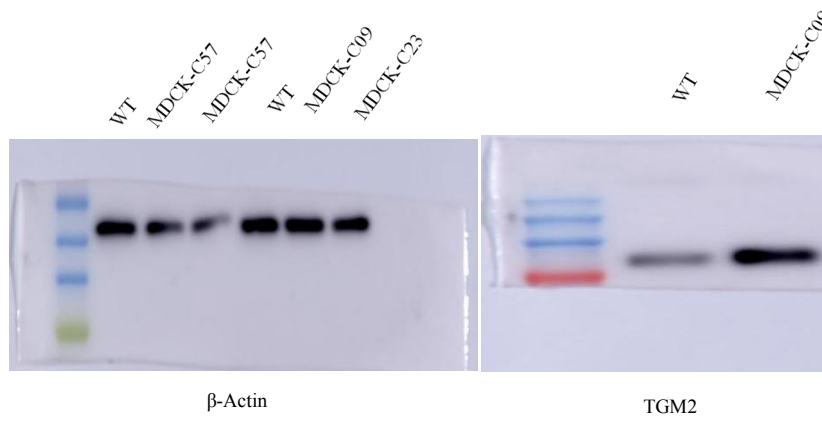

Fig2

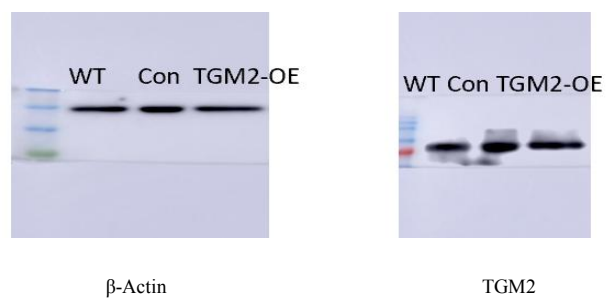

Fig3

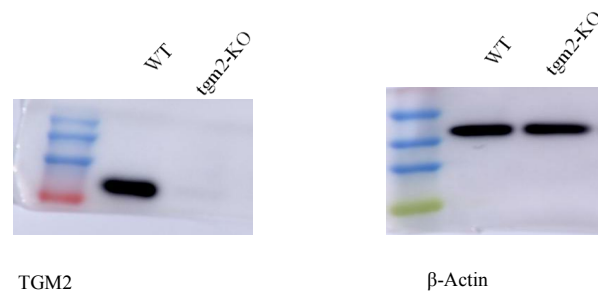

Fig4

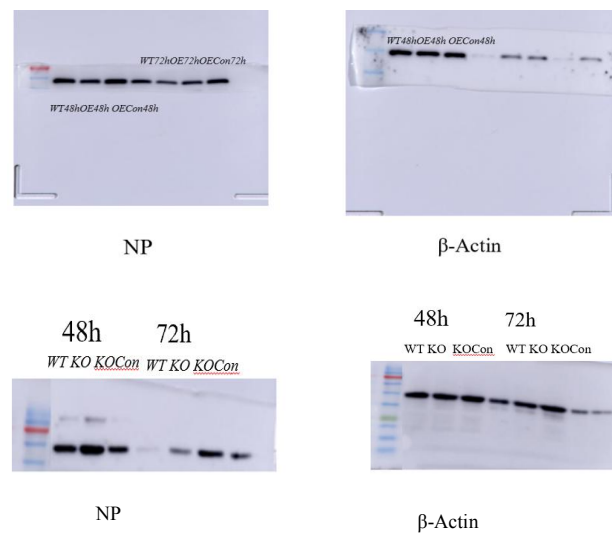

Fig6

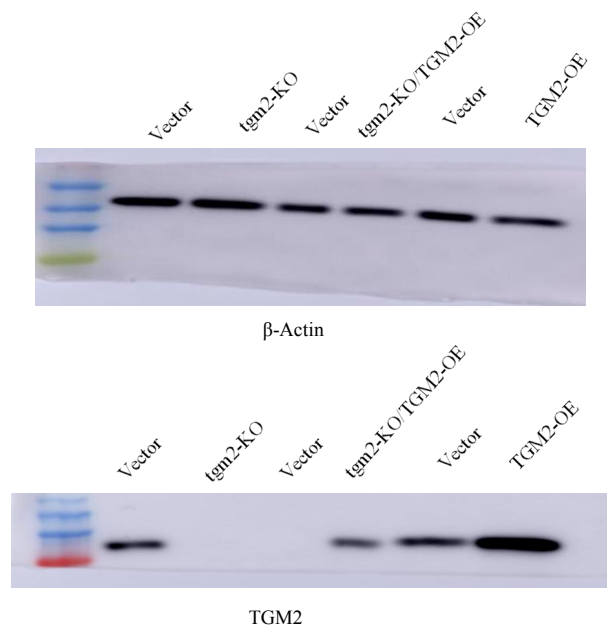

Fig7

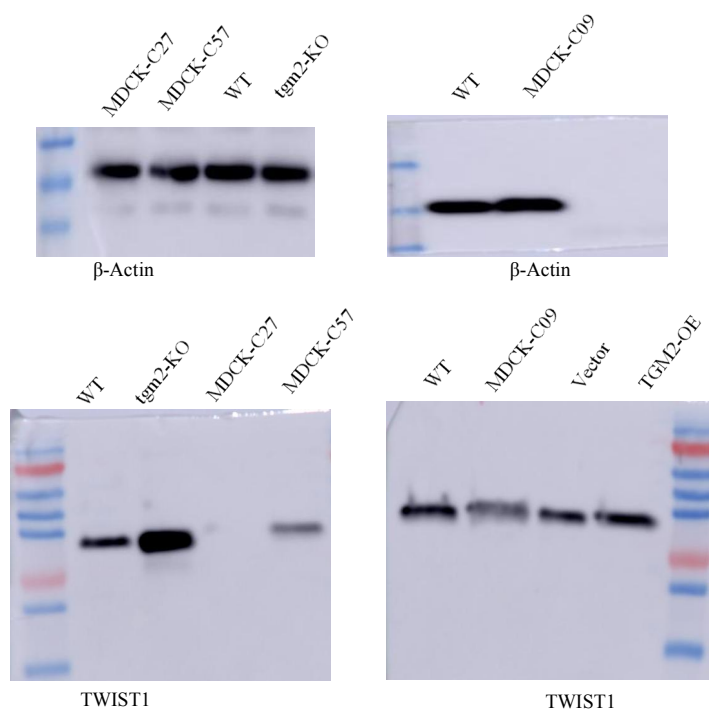

Supplement: S2 File — (PDF) [file pone.0285136.s002.pdf]
