# Supplementary material for: TGM2 inhibits the proliferation, migration and tumorigenesis of MDCK cells
Source: PLoS One. 2023 Apr 28;18(4):e0285136. doi: 10.1371/journal.pone.0285136 (PMC10146566; doi:10.1371/journal.pone.0285136)

Fig.S1: Detection of cell cycle by flow cytometry in TGM2 overexpressing cells and vector control cells. (A) Cell cycle of vector control cells. (B) Cell cycle of TGM2 overexpressing cells.

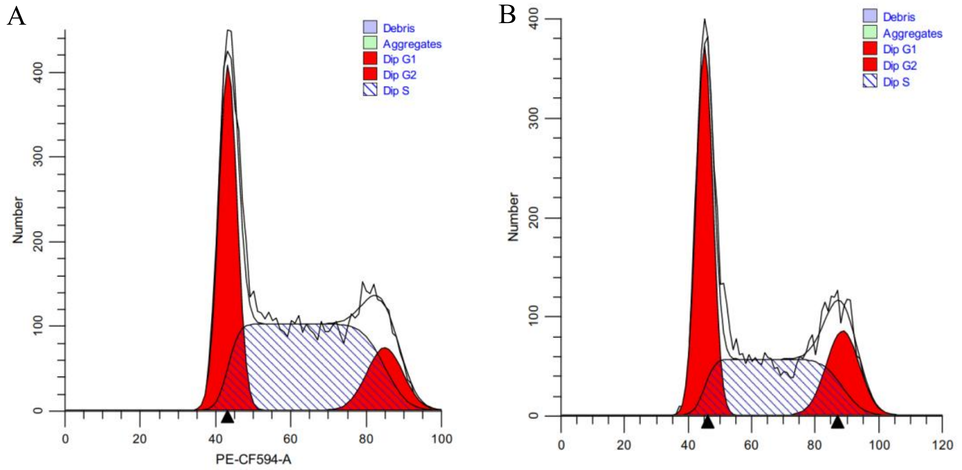

Fig.S2: Detection of cell cycle by flow cytometry in TGM2-knockout cells and Wild-type cells. (A) Cell cycle of Wild-type cells. (B) Cell cycle of TGM2-knockout cells.

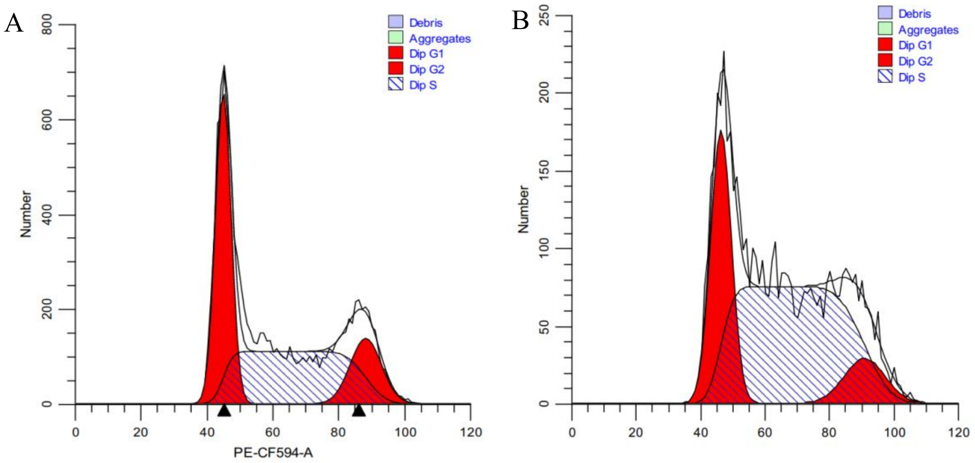

Supplement: S3 File — (PDF) [file pone.0285136.s003.pdf]
